# Complex functional and epithetic rehabilitation after ablation of recurrent retroauricular basal cell carcinoma – a case study

**DOI:** 10.3205/iprs000120

**Published:** 2017-12-18

**Authors:** Waldemar Reich, Anika Exner, Eileen Winter, Bilal Al-Nawas, Alexander Walter Eckert

**Affiliations:** 1 Department of Oral and Plastic Maxillofacial Surgery, Martin Luther University Halle-Wittenberg, Halle (Saale), Germany; 2 University School of Dental Medicine, Martin Luther University Halle-Wittenberg, Halle (Saale), Germany

**Keywords:** auricular, basal cell carcinoma, concha, craniofacial, epithesis, implant, insertion side, mastoid, radiotherapy, tumor resection

## Abstract

The reconstruction of extended defects of the concha poses a complex challenge for plastic surgeons. In cases of subtotal ablation, an alternative method designed especially for elderly oncological patients consists of epithetic rehabilitation. However, inserting an implant-retained concha epithesis proves challenging in patients with antecedents of deep resections involving the mastoid process.

In the present case study, we report on the long-term treatment course (2009–2017) of a 79-year-old male patient suffering from a recurrent basal cell carcinoma of the retroauricular region. Following tumor resection, along with lateral mastoidectomy, reconstruction, and adjuvant radiotherapy, functional and esthetic deficits primarily due to peripheral facial nerve palsy were successfully managed using a multistep procedure. The procedure was completed by inserting an implant-retained concha epithesis, resulting in improved quality of life. Due to prior lateral mastoidectomy, ultra-short implants (4 mm) were inserted, partially at atypical positions. For maintaining healthy periimplant soft tissue, aftercare comprised cold plasma treatment.

This oncologic case demonstrates the therapeutic necessity of using a broad spectrum of reconstructive procedures, along with their limitations, in a critical anatomic region. Specific features include the presentation of a workflow using ultra-short implants in a compromised mastoid region. Surgeons should consider alternative implant positions in the event of any compromised mastoid process. A particular emphasis has been put on meticulous aftercare to preserve healthy periimplant soft tissues.

## Zusammenfassung

Die Rekonstruktion größerer Ohrmuscheldefekte stellt eine komplexe plastisch-chirurgische Herausforderung dar. Eine etablierte Alternative bei subtotalen Defekten, insbesondere bei älteren onkologischen Patienten, besteht in der epithetischen Rehabilitation. Die implantologische Verankerung einer Ohrepithese ist jedoch anspruchsvoll im Falle einer vorhergehenden tiefen Resektion, welche den Procesus mastoideus erfasst.

Der vorliegende Fallbericht stellt den langfristigen Behandlungsverlauf (2009–2017) eines männlichen 79-jährigen Patienten dar, der an einem rezidivierenden retroaurikulären Basalzellkarzinoms rechts litt. Nach der Tumorresektion einschließlich einer lateralen Mastoidektomie, Rekonstruktion und adjuvanter Strahlentherapie folgten weitere funktionell-ästhetische Korrekturen bei peripherer Fazialisparese. Abschließend wurde eine implantat-getragene Ohrepithese hergestellt, welche wesentlich zur Verbesserung der Lebensqualität beitrug. Aufgrund der vorhergehenden lateralen Mastoidektomie mussten ultra-kurze Implantate (4 mm) verwendet und zum Teil an atypischer Position inseriert werden. In der Nachsorge der periimplantären Weichteile kam kaltes Atmosphärenplasma zur Anwendung.

Der onkologische Fallbericht verdeutlicht die therapeutische Notwendigkeit eines breiten rekonstruktiven Spektrums und deren Limitationen in einer kritischen anatomischen Region. Besonderheiten dieses Beitrags bestehen in der Präsentation eines Behandlungsprotokolls unter Verwendung ultra-kurzer Implantate in einer kompromittierten Mastoidregion, wobei alternative Implantatpositionen berücksichtigt worden sind. Eine sorgfältige Nachsorge zur Aufrechterhaltung gesunder periimplantärer Weichteile ist hervorgehoben.

## Introduction

The facial skin may be affected by several malignant tumors. Among all known non-melanotic skin cancers, the basal cell carcinoma is predominant, involving 80% of all cases. Furthermore, squamous cell carcinomas, merkel cell carcinomas, adnex carcinomas, as well as others, require surgical treatment [1]. Depending on the morphological subtype, micrographic surgical treatment of basal cell carcinomas requires safety margins of at least 3 to more than 10 mm [1], [2]. After partial ablation of the concha, excellent esthetic results may be achieved by means of an artful realisation of various local flaps [2], [3].

In the event of (sub)total or extensive resections, along with adjuvant radiotherapy, local preconditions for plastic rehabilitation and external ear reconstruction prove challenging, especially when involving the facial nerve and meatus acusticus externus. Therefore, epithetic rehabilitation presents a valuable treatment option in the whole treatment course for elderly oncological patients [4], with implant fixation preferred in 92% of cases [5]. 

Deep resections and adjuvant radiotherapies result in limited bone volume and vulnerable tissues, which require optimal preoperative planning and meticulous postoperative follow-up. Furthermore, a particular anatomical feature pertaining to the auricular bone-anchored epithesis consists in a distinct range of motion of the mandible and head [6].

When considering the origin of dental implantology, biological preconditions for craniofacial implants were already established 30 years ago. Concerning recent additional treatment protocols for limited bone quantity using *short dental* implants [7], it must be emphasized that in terms of macro-design, *short craniofacial* screw implants had been successfully employed for a longer period [8], [9].

In the present case study, we describe a precise workflow using ultra-short craniofacial implants, and this in a compromised mastoidal region.

## Case presentation

### Medical history

In 2009, a male 79-year-old patient was initially referred to our center due to a persisting retroauricular node. The reported comorbidities have been detailed in Table 1 (Tab. 1). His psychosocial environment was satisfactory. The physical examination was performed by an oral and maxillofacial surgeon. Except for the painless nodular efflorescence in the retroauricular area, no other anomalies were found in the head and neck region, and the neurologic examination was normal. The patient presented a skin type II. Clinical and histhopathological examination confirmed the presence of a nodular basal cell carcinoma (ICD-10 code: C44.2), which was resected in sano under local anesthesia. The skin was reconstructed by a Limberg-flap without tension, the healing period and later follow-up examinations being totally uneventful. 

**Figure 1:**
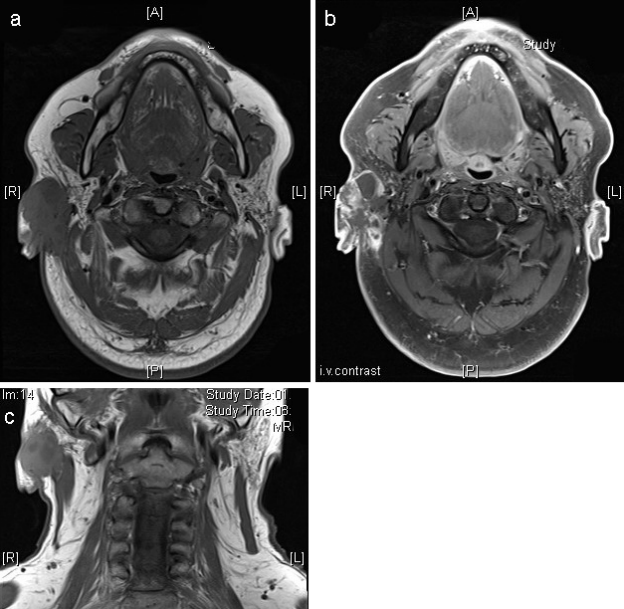
a: Preoperative magnetic resonance imaging (MRI) – axial section (tra tse t1). The hypointense tumor represents a mass of 47.5x43x34.6 mm that infiltrates the caudal meatus acusticus externus and the parotid gland, attaining up to the sternocleidomastoid and nuchal muscles (rT4N1M0). b: Preoperative MRI – axial section (tra tse t1 with contrast agent). The tumor is characterized by inhomogeneous cysteiform contrast agent uptake. c: Preoperative MRI – coronal section (tse t1)

**Figure 2:**
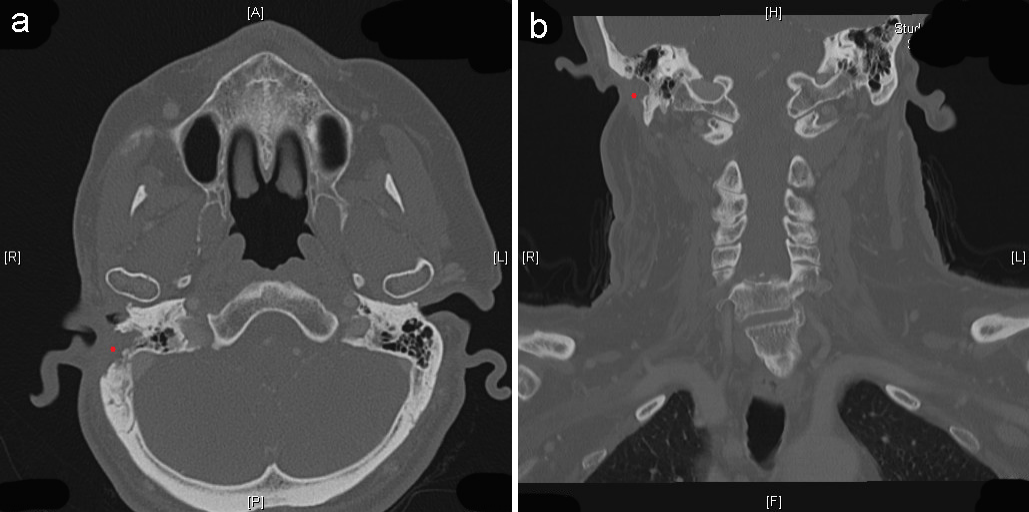
a: Follow-up imaging – axial computerized tomography (CT) section. Red asterisk indicating the partially resected right mastoid process. b: Follow-up imaging – coronal CT section. Red asterisk indicating the partially resected right mastoid process.

Nevertheless, three years later, the patient returned to the clinic with a recurrent retroauricular swelling, which infiltrated the sternocleidomastoideous muscle and caudally the external meatus acusticus. Preoperative audiometry revealed a basocochlear labyrinthine hearing loss (presbyacusis). Following magnetic resonance imaging (MRI) (Figure 1 (Fig. 1)), histopathological confirmation, and multidisciplinary discussion of treatment options, a radical tumor resection was performed. The procedures consisted of a subtotal concha ablation, radical parotidectomia, as well as removal of caudal part of the external meatus acusticus and cranial part of the sternocleidomastoideus muscle as en-bloc resection. Additionally, for the purpose of evaluating safety margins, a lateral mastoidectomia was deemed necessary (Dr. M. Herzog is acknowledged; follow-up computer tomography [CT] in Figure 2 (Fig. 2)). Owing to peripheral facial nerve palsy, a lateral canthoplasty was simultaneously performed [10]. On account of an 8x9 cm resection defect, an ipsilateral pedicled myocutaneous latissimus-dorsi flap was raised. The postoperative period was uneventful, and following primary wound healing, adjuvant radiotherapy ad 64Gy was applied. 

In Table 1 (Tab. 1), a detailed synopsis of the whole treatment course has been presented, comprising among others eye brow lifting, upper eyelid loading (1.2 g platin chain, Spiggle & Theis, Overath, Germany), and elevation of the paralytic angulus oris by a facia lata sling. Figure 1 (Fig. 1) illustrates the clinical status of the auricular region prior to initiating implantological treatment. 

### Implantological treatment

**Figure 3:**
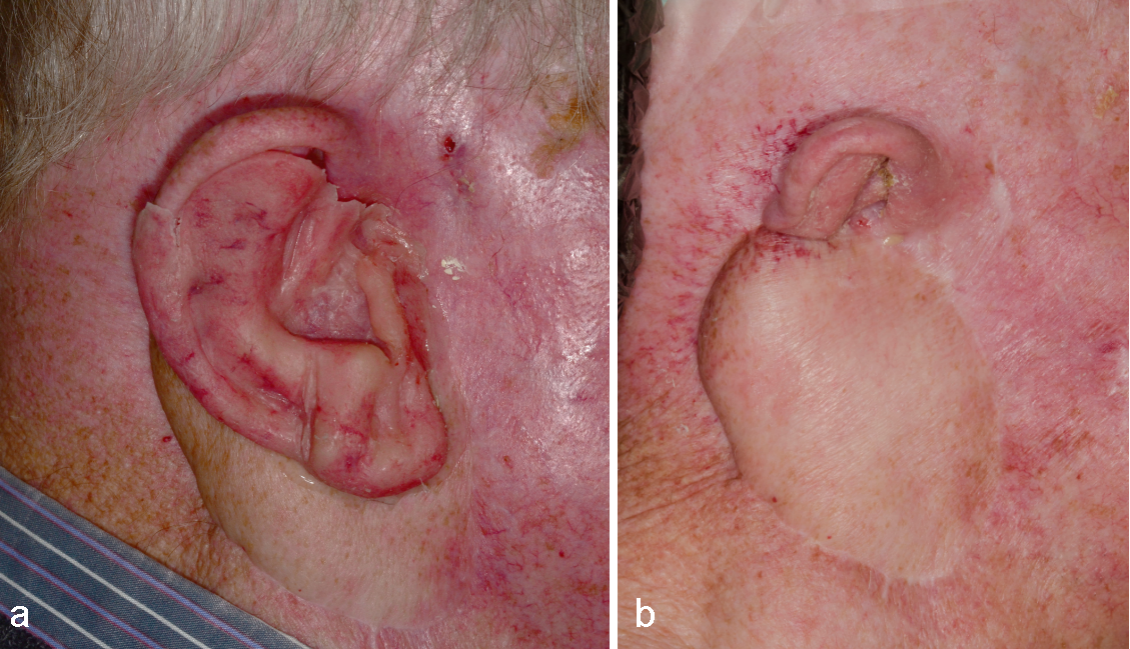
a: Adhesive retained interim epithesis. Note the preauricular actinic keratosis and scarification after removal (Figure b). b: Lateral view of the right auricular region prior to definitive epithetic treatment. The regional soft tissue is characterized by radioderm, stenosis of the external porus acusticus, residual concha, and voluminous myocutaneous flap.

**Figure 4:**
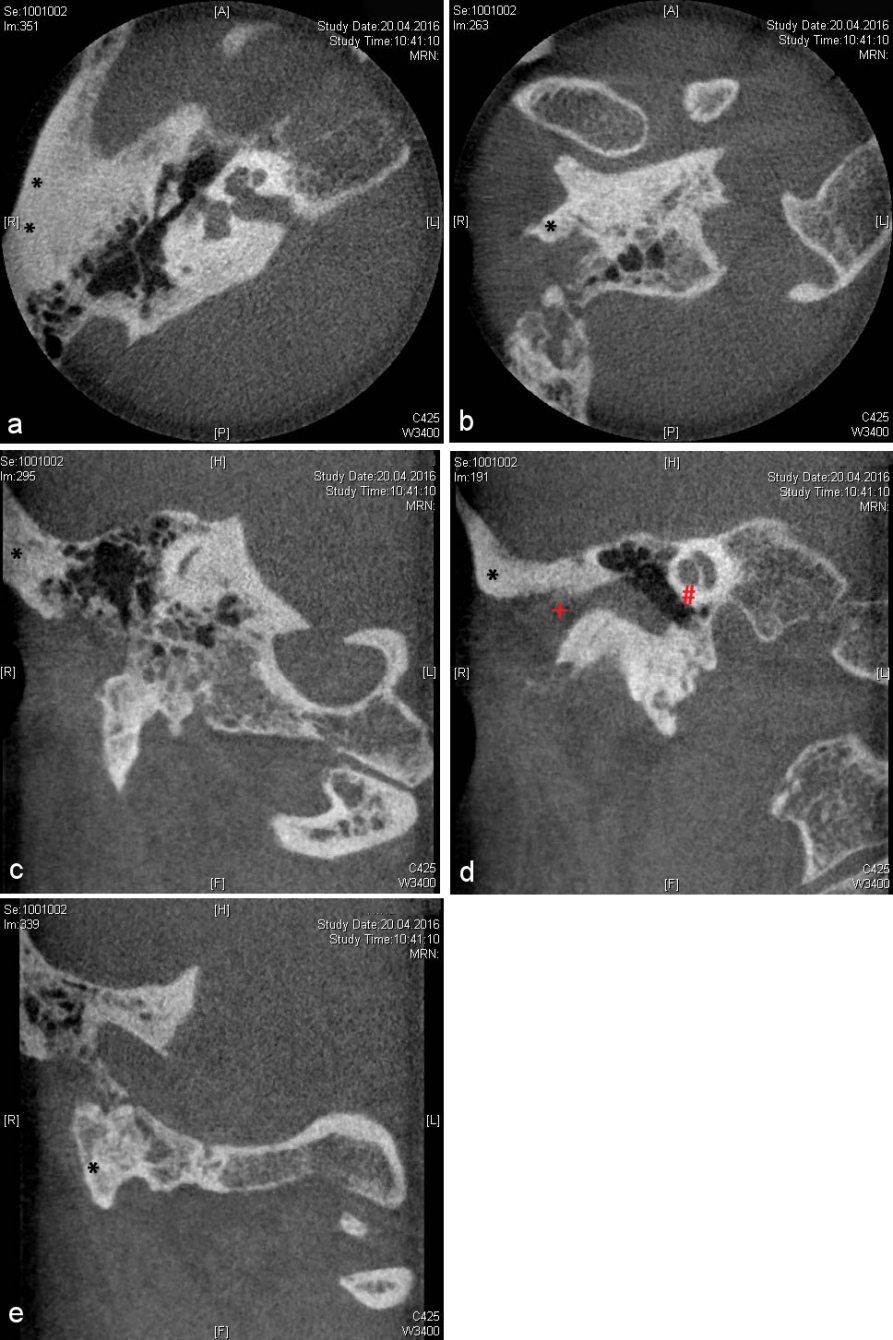
Preoperative cone beam computerized tomography (CBCT) imaging. a: Axial section. Black asterisk indicating planned cranial implant positions. b: Axial section. Black asterisk indicating planned caudal implant position. c: Coronal section. Black asterisk indicating planned first cranial implant position. d: Coronal section. Black asterisk indicating planned second cranial implant position, red plus marking the external meatus acusticus, and red diamond showing the cochlea. e: Coronal section. Black asterisk indicating planned caudal implant position.

Following a recurrence free interval, the patient was supplied with a temporary adhesive-retained epithesis (B-400 Secure medical adhesive, Daro B-200-30 adhesive, Cosmesil, Heidelberg, Germany). This epithesis covered the residual cranial concha, thereby providing undercuts and an additional retentive surface (Figure 3 (Fig. 3)). The definitive epithetic treatment was then conducted following 3D preoperative assessment involving cone beam computerized tomography (CBCT) of the temporal bone (Figure 4 (Fig. 4)).

**Figure 5:**
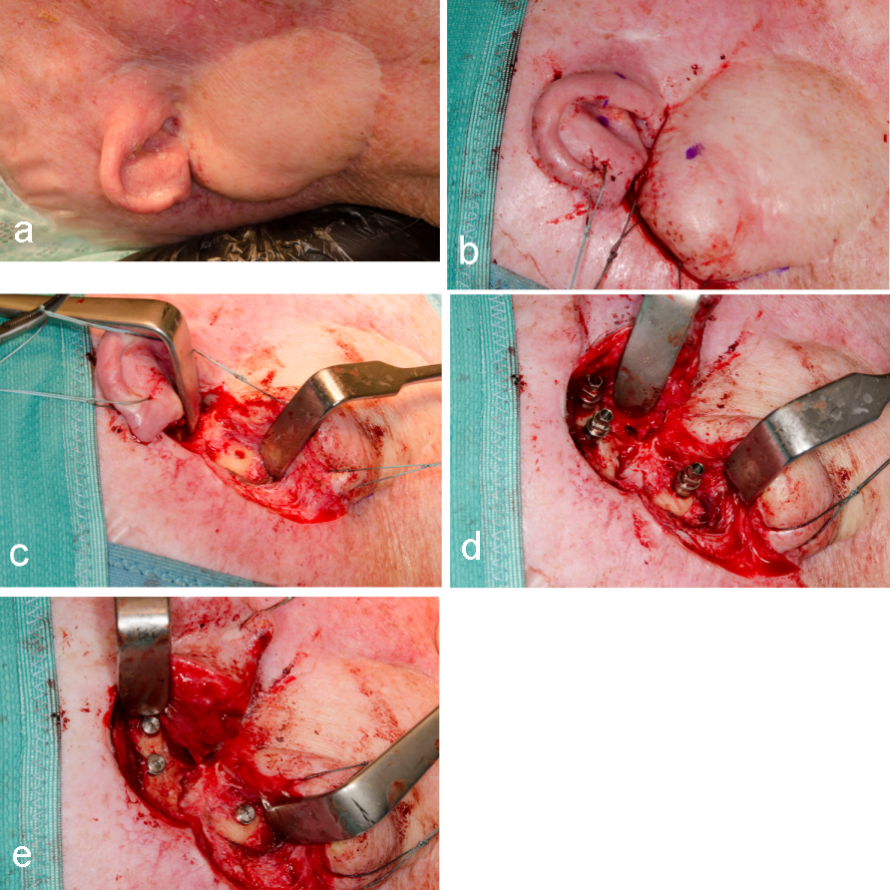
a Preoperative clinical view of the auricular region. b: Intraoperative view of surgical access route. Blue dots marking the intended implant positions. c: Intraoperative view of the prepared caudal implant bed. Fresh bleeding demonstrating vital bone of the residual mastoid process. d: Intraoperative view of inserted implants parallel to each other. e: Intraoperative view after removal of the insertion pins and fixation of cover screws owing to closed healing.

**Figure 6:**
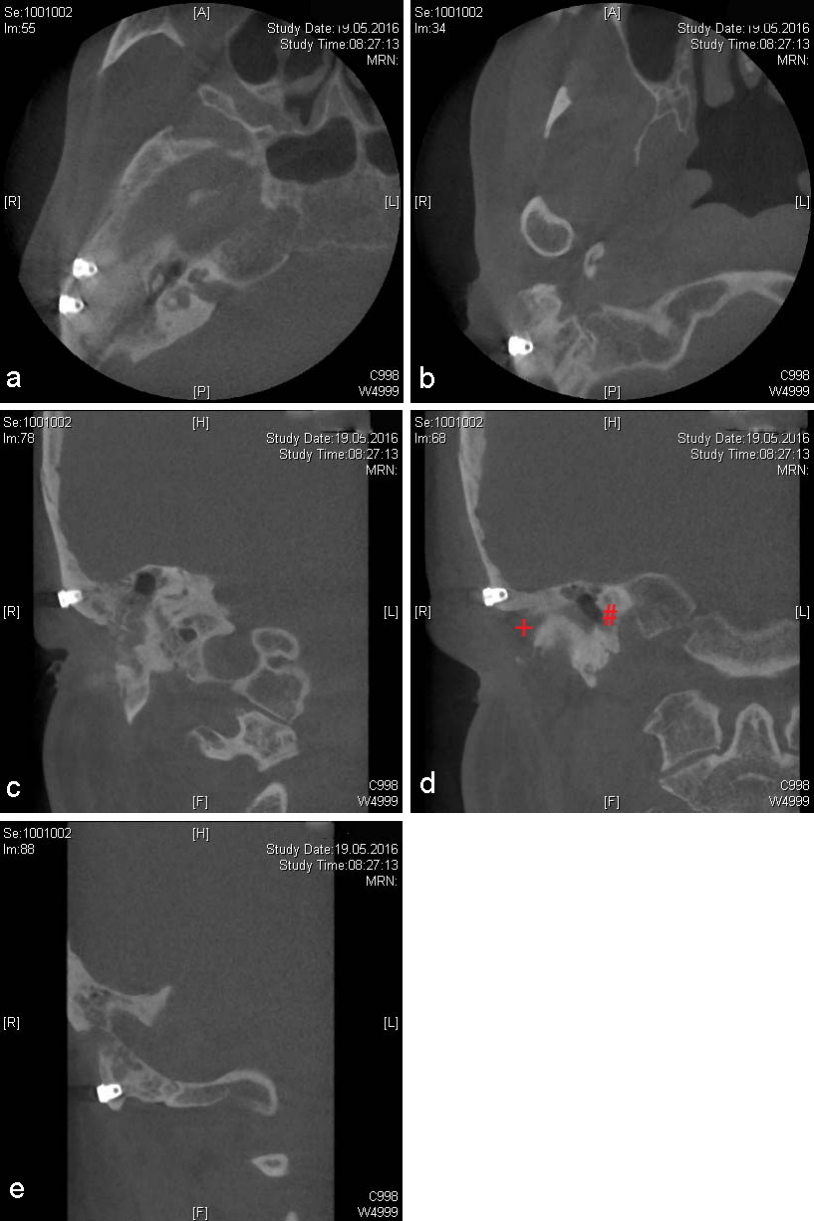
Postoperative CBCT-imaging. a: Axial section. Both cranial implant positions. b: Axial section. Caudal implant position. c: Coronal section. First cranial implant position. d: Coronal section. Second cranial implant position, red plus marking the external meatus acusticus, and red diamond indicating the cochlea. e: Coronal section. Caudal implant position.

Under general anesthesia and including perioperative systemic antibiotic treatment (Cefuroxim 2x 500 mg daily per os), three endosseous screw implants [11] were inserted (Southern implants, 4x3.75 mm, Gauteng, South Africa). A duplicate of the interim epithesis was used as a drilling splint, based on the preoperative planing (Figure 5 (Fig. 5)). Two implants were inserted cranialy to the external meatus acusticus, and the remaining one in the residual mastoid process, thus avoiding pneumatized areas (Figure 6 (Fig. 6)). The healing period proved uneventful.

**Figure 7:**
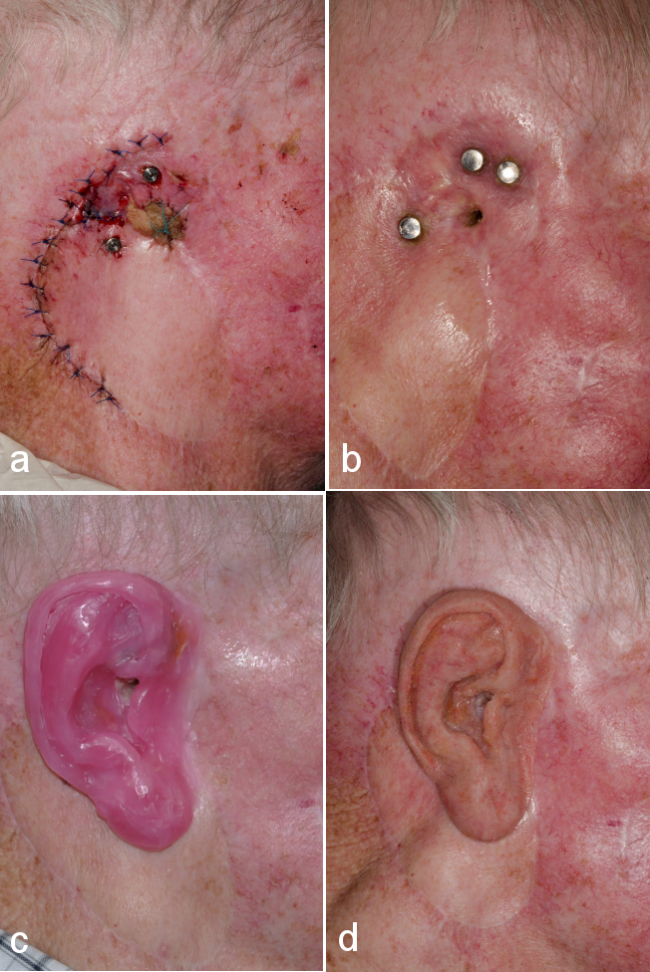
a: Postoperative view after the re-entry operation. The residual concha was resected, with the stenosis of the external porus acusticus additionally corrected by a rotation flap and full-thickness skin graft (concha). b: Lateral view of the auricular region before referral to the anaplastologist. As can be seen, the stenosis has been managed successfully, the magnetic abutments have been inserted, with the soft tissue conditioning completed following repeated removal of incrustations. c: Lateral view after precise wax modelation was completed. The actual implant positions are considered totally satisfactory. d: Lateral view of the completed implant-retained concha epithesis.

All three ultra-short osseo-integrated implants were regularly followed-up, with the re-entry operation scheduled 4 months later. During this session, the stenosis of the external porus acusticus was managed using a local rotation flap and full-thickness skin graft from the resected remaining concha (Figure 7a (Fig. 7)). The intraoperative otoscopia revealed no relevant pathology. At this time, in an effort to preserve perfusion, the adjacent periimplant skin was not thinned out definitely, which was performed at a later time, under local anesthesia. The soft tisue conditioning was addressed by inserting healing abutments of 8 mm height. 

Six months after implantation [11], [12], we were able to insert definitive magnetic abutments (2 Z-line, 1 T-Line, Steco, Hamburg, Germany), and the patient was referred to the anaplastologist (Figure 7b (Fig. 7)). The magnetic inserts could easily be removed by a special applicator for cleansing and MRI diagnostics. 

### Manufacturing of the concha epithesis

The impression was taken using flowable silicon (Stecoflex, steco-system-technik, Hamburg, Germany), which was stabilized by means of wood spatulas and a firm silicon (Multisil hard-form, Bredent medical, Senden, Germany). Thereafter, wax modellation was performed, which was followed by photo-technical measurement and individualisation (Figure 7c (Fig. 7)). Thereafter, the casting mold could be manufactured using super-hard gypsum. The manufacturing process (Cosmesil 551, Cosmesil, Heidelberg, Germany) was completed using intrinsic coloration and layered arrangement of the medical silicon. The completed epithesis has been presented in Figure 7d (Fig. 7). For functional reasons, the external porus acusticus was spared out. For creating an adaptable anterior margin across the complete range of head and mandibular motion, the technique described by Kubon (2001) can be considered [6].

### Follow-up

The patient was instructed to meet the recommendations for (daily) care [13] and recall examinations as follows:

- clean with lukewarm water, mild soap, and hand/tooth brush
- at night, remove the epithesis for the sake of skin regeneration
- silicon material has a limited “life span”, with limited possibilities of repair
- avoid sun exposition, as this leads to color differences between the epithesis and adjacent skin
- prior to any MRI, obligatorily remove magnetic abutments using an applicator
- attend quarterly clinical follow-up for oncological reasons, care of both periimplant soft tissue (antiseptic and decontamination by cold plama treatment; plasma ONE, Plasma medical systems GmbH, Bad Ems, Germany [14], [15]) and external meatus acusticus, as well as ophthalmologic examinations, which are all essential for long-term success.

The patient reported a significant improvement in his health-related quality of life.

Lastly, it should be noted that in the course of clinical follow-up, further actinic keratoses of the facial skin (nasal dorsum, cheeck, infraorbital region) and another basal cell carcinoma of the contralateral concha were observed, requiring surgical treatment.

## Discussion

Implant-retained craniofacial epitheses represent a suitable therapeutic alternative for various congenital or acquired conditions involving the auricular region [11], [16]. Recently, advanced computer-aided planning, design, and manufacturing have been instrumental in achieving predictable treatment results [17]. When deciding on the adequate fixation of an auricular epithesis, the patient age, expectations, and life style are essential criteria to be taken into account [13]. In addition to bone-anchored fixation, which is the standard method, using skin adhesives appears appropriate under certain conditions [18]. This kind of retention is indicated for elderly patients [5], [19], and particularly for the purpose of temporary supply, as initially performed in our case. 

In Table 2 (Tab. 2), advantages and disadvantages of both retentive options have been summarized. Significant disadvantages to using skin adhesives include margin damage of the epithesis by repeated application and removal, potential toxic skin reaction, insufficient retentive capacity in mobile tissues like temporomandibular joint, or presence of hair [18]. In the present case, over time, the patient complained of increasing retention loss of the adhesive-retained epithesis due to vulnerability of the irradiated soft tissues, and of ptosis of the remained concha. Three years following oncological pretreatment, a surgical intervention was deemed unavoidable, due to a slowly progredient re-stenoisis of the external meatus acusticus. Only at that time, the elderly patient provided his consent to undergo implantation.

Concerning the individual choice of the abutment type in bone-supported epitheses, magnetic devices bear several advantages over bar/plate devices, namely flat construction of the epithesis with thin margins, esthetical appearance, better periimplant cleanability, and low periimplant biomechanical stress due to repeated application and removal [12], [19]. 

Concerning the prognosis of craniofacial implants according to the anatomical region, a recent systematic literature review by Chrcanovic et al. (2016) (n=8184 implants in 2355 patients) revealed the following implant failure probabilities: 1.2% in the auricular region, 12.1% in the orbital region, and 12.2% in the nasal region [4]. Furthermore, the authors demonstrated radiotherapy to significantly affect the fate of craniofacial implants (OR 5.8). In an earlier monocentric retrospective study (n=150 implants in 56 patients), the authors reported comparable 2-year survival rates: 94.1% for auricular implants, 90.9% for nasal implants, 100% for orbital implants, and 100% for complex midfacial implants [20].

It should be noted that implant insertion in the auricular region proves technically challenging [21], owing to limited native bone, especially in the event of prior bone resection (Figure 2 (Fig. 2)), as well as soft tissue deformity (Figure 3a (Fig. 3)). Therefore, image-guided placement of implants appears obligatory [9]. In brief, in the auricular region, it appears mandatory to insert at least two implants in the mastoid process (8–9 o’clock position) in a distance of 2 cm from the external meatus acusticus [12]. Due to previous lateral mastoidectomy (Table 1 (Tab. 1)), we inserted three implants in a triangular arrangement (9–12–1 o’clock position), whilst deviating from the ideal position. Of note is that this issue was carefully discussed prior to the intervention with the anaplastologist, based on the 3D-imaging data. As shown in Figures 7c–d (Fig. 7), no extension of the epithesis in the antero-cranial direction was required. In contrary, the triangular implant position was prospectively deemed a biomechanical advantage with respect to the force distribution within the limited bone area (surrounding ultra-short implants) upon repeated epithesis insertion and removal [12]. Additionally, Xing et al. confirmed for the orbital region (2017) that an increase in implant diameter does significantly more decrease biomechanical periimplant stress, as compared to an increase in implant length [22].

Literature search (PubMed) using the terms “craniofacial implants” AND “temporal bone” OR “mastoid bone” AND “insertion side” did not display any relevant results. On account of this, we were unable to compare our modified insertion sides to previously published experiences. 

When thinning out the periimplant soft tissue, special care has to be taken as to the following constraints: a) sufficiently remove adjacent fibers of the temporal muscle; b) avoid an accidental incision of the temporomandibular joint capsula (pars temporalis). Upon long-term follow-up, there were no clinical signs of limited jaw movement, in spite of the fixed periimplant soft tissue (see movie clip in Attachment 1).

According to Wagenblast et al. (2008), psychosocial features of caniofacial epitheses are paramount [23]. The flexibility of silicon and low termal conductivity enable high wearing comfort, with the epithesis considered to be “part” of the body. These results have been confirmed by our presented case, as well as by the publication from Zuo and Wikes (2016) [21]. Compared to other localisations, auricular epitheses were assessed as the most comfortable solution by the patient [24].

## Conclusions

Based on the present case study, it can be concluded that extended concha ablation due to skin malignancies requires a broad spectrum of reconstructive procedures. Plastic surgeons are faced with compromised hard and soft tissues pertaining to this critical anatomic region, which is especially true for elderly irradiated patients. In the event of a compromised mastoid process, clinicians may consider the dorsal zygomatic process of the temporal bone as an alternative recipient side for auricular craniofacial implants. Should this be the case, the bone-anchored epithetic rehabilitation using both ultra-short implants and magnetic abutments is deemed a valuable and safe treatment option, yet requiring regular multidisciplinary aftercare. 

## List of abbreviations

- CBCT = Cone beam computerized tomography
- CT = Computerized tomography
- Gy = Gray
- ICD-10 = International Classification of Diseases, Version 10
- MRI = Magnetic resonance imaging
- RT = Radiotherapy
- rT2N1M0 = Tumor staging

## Notes

### Competing interests

WR, AE, EW, and AWE declare that they have no competing interests. BA is providing lectures for Straumann, Camlog, Dentsply, Nobel biocare, Geistlich.

### Ethical approval and consent to participate

The patient signed the informed consent form. The procedures performed in this case study were in accordance with ethical standards of the 1964 Helsinki Declaration and its subsequent amendments.

### Consent for publication

Written informed consent for publication of clinical data and clinical images was obtained from the patient. A copy of the written consent form is available on request for review by the journal’s editor. 

### Authors' contributions

WR: Performed the surgical treatment and aftercare, and contributed to drafting the manuscript.

AE: Drafted the article.

EW: Analyzed the 3D-imaging and searched for relevant literature.

BA: Critically revised the article for intellectual editorial content.

AE: Analyzed and interpreted the patient data regarding the surgical treatments.

All authors have read and approved the final version of the manuscript.

### Acknowledgements

The authors would like to thank PD Dr. M. Herzog (Department of Laryngo-Rhino-Otology, Head and Neck Surgery, University Hospital Halle, Germany; since recently, at Carl-Thiem Hospital Cottbus, Department of Laryngo-Rhino-Otology, Head and Neck Surgery, Germany) for performing the lateral mastoidectomy, and Sylvia Dehnbostel (Institute for Anaplastology, Celle, Germany) for manufacturing the epithesis. In addition, we acknowledge Dr. Gabrielle Cremer Consulting (https://cremerconsulting.com) for providing English language editing.

## Supplementary Material

Movie clip: Behavior of periimplant soft tissues under maximal mouth opening

## Figures and Tables

**Table 1:**
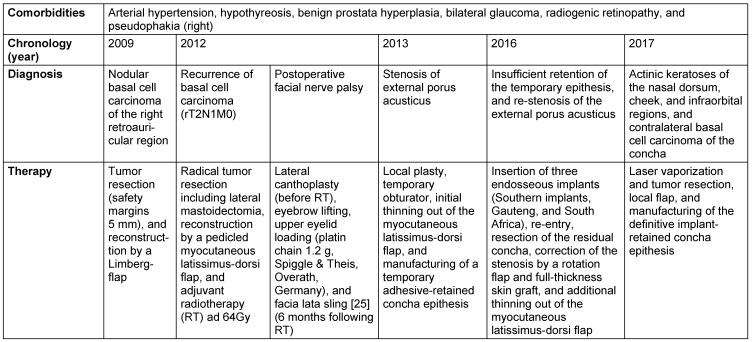
Treatment course synopsis

**Table 2:**
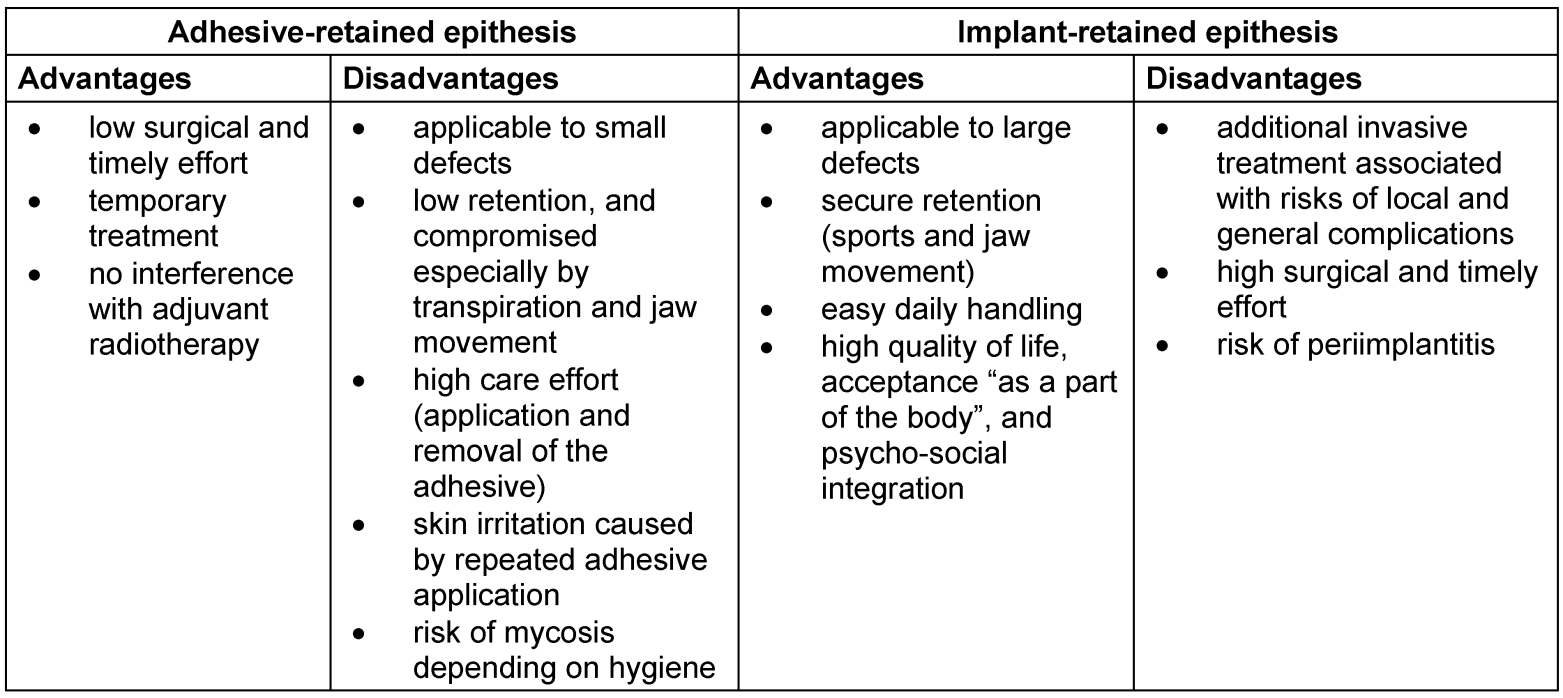
Comparison of the adhesive-retained vs. implant-retained epitheses (modified according to [13])
